# Supplementary material for: Targeting Adaptive IRE1α Signaling and PLK2 in Multiple Myeloma: Possible Anti-Tumor Mechanisms of KIRA8 and Nilotinib
Source: Int J Mol Sci. 2020 Aug 31;21(17):6314. doi: 10.3390/ijms21176314 (PMC7504392; doi:10.3390/ijms21176314)
Supplement: Supplementary file 1 [file ijms-21-06314-s001.zip › Supplementary Materials/Yamashita Table S2.docx]

**Table S2. qRT-PCR primer sequences**

| Gene | Forwards 5’→3’ | Reverse 5’→3’ |
| --- | --- | --- |
| *ACTB* | AGAGCTACGAGCTGCCTGAC | AGCACTGTGTTGGCGTACAG |
| *CHOP* | AGAGCTACGAGCTGCCTGAC | AGCACTGTGTTGGCGTACAG |
| *PLK1* | GACACTGCAACCAACACCAC | GTCTCTGGCTCCGTGATAGC |
| *PLK2* | GAGCAGCTGAGCACATCATT | CATGTGAGCACCATTGTTGA |
| *sXBP1* | GAGTCCGCAGCAGGTG | TCCTTCTGGGTA GACCTCTGGGAG |
| *BIP* | TAGCGTATGGTGCTGCTGTC | TTTGTCAGGGGTCTTTCACC |
| *ATF4* | AAGGCGGGCTCCTCCGAATGG | CAATCTGTCCCGGAGAAGGCATCC |
| *ATF6* | ACCTGCTGTTACCAGCTACCACCCA | GCATCATCACTTCGTAGTCCTGCCC |
| *TXNIP* | CCTCTGGGAACATCCTTCAA | GGGGTATTGACATCCACCAG |
| *Rat Actβ* | GCAAATGCTTCTAGGCGGAC | AAGAAAGGGTGTAAAACGCAGC |
| *Rat Plk1* | CAGGAAGCCTCTCACAGTCC | CCCAGAAGATAGGGATGCAA |
| *Rat Plk2* | ATCCGCAGTGGAAAACAAAC | CACTGAAAGGACGTGCTCAA |
