# Supplementary material for: Targeting Adaptive IRE1α Signaling and PLK2 in Multiple Myeloma: Possible Anti-Tumor Mechanisms of KIRA8 and Nilotinib
Source: Int J Mol Sci. 2020 Aug 31;21(17):6314. doi: 10.3390/ijms21176314 (PMC7504392; doi:10.3390/ijms21176314)
Supplement: Supplementary file 1 [file ijms-21-06314-s001.zip › Supplementary Materials/Yamashita Figure S1.docx]

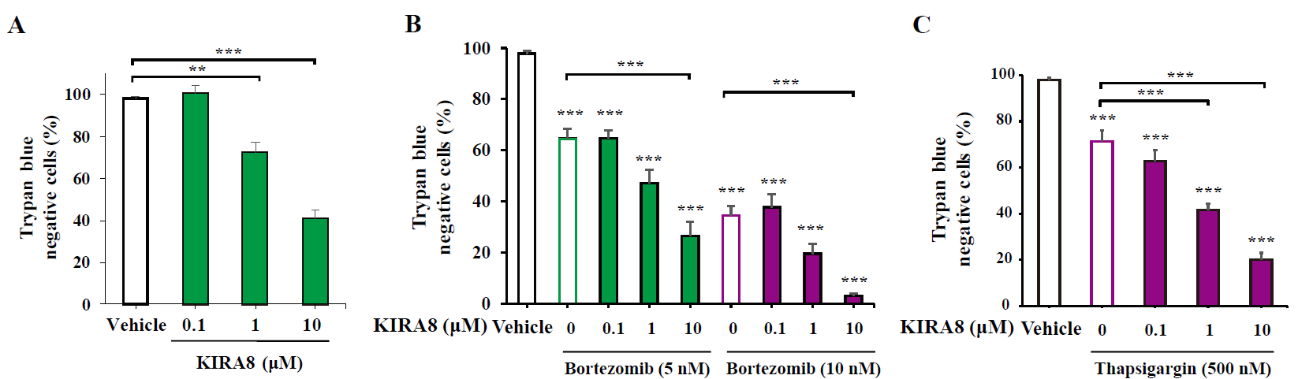


**Supplementary Figure 1. The trypan blue exclusion assay shows a significant cell viability reduction in human myeloma cells.** (a) IM-9 cells were treated with vehicle (DMSO) or KIRA8 (at 0·1, 1, and 10 μM; b and c) for 1 h, followed by bortezomib (5 nM or 10 nM) of or 500 nM of thapsigargin for 24 h. The cell viability was confirmed by the trypan blue exclusion assay. Data shown are mean ± SEM. For these experiments, six independent biological samples were used.
