# Supplementary material for: Targeting Adaptive IRE1α Signaling and PLK2 in Multiple Myeloma: Possible Anti-Tumor Mechanisms of KIRA8 and Nilotinib
Source: Int J Mol Sci. 2020 Aug 31;21(17):6314. doi: 10.3390/ijms21176314 (PMC7504392; doi:10.3390/ijms21176314)
Supplement: Supplementary file 1 [file ijms-21-06314-s001.zip › Supplementary Materials/Yamashita Table S1.docx]

**Table S1. Clinical characteristics of our patients in this study**

| Case | Sex | Age (years) | NCC (x10^4^/μL)* | Myeloma % | First treatment (Outcome) |
| --- | --- | --- | --- | --- | --- |
| MM-1 | F | 72 | 13.5 | 18.4 | Bortezomib-based regimen → HD-MEL (VGPR) |
| MM-2 | F | 77 | 6.5 | 45.2 | Lenalidomide-based regimen (PD) |
| MM-3 | M | 48 | 43.8 | 74.4 | Bortezomib-based regimen → HD-MEL (CR) |
| MM-4 | M | 67 | 6.0 | 42.4 | Bortezomib-based regimen (CR, but complicated CKD) |
| MM-5 | M | 70 | 10.9 | 25.6 | Bortezomib-based regimen → HD-MEL (VGPR) |
| MM-6 | F | 57 | 19.9 | 54 | Bortezomib-based regimen → HD-MEL (CR) |
| MM-7 | M | 43 | 10.2 | 19.6 | Bortezomib-based regimen → HD-MEL (CR) |
| MM-8 | M | 59 | 11.5 | 42.8 | Bortezomib-based regimen → Lenalidomide-based regimen (PD) |
| MM-9 | F | 52 | 4.7 | 56 | Bortezomib-based regimen → HD-MEL (CR) |
| MM-10 | M | 56 | 11.2 | 64.8 | Bortezomib-based regimen → HD-MEL (CR) |
| MM-11 | M | 69 | 8.1 | 17.4 | Bortezomib-based regimen → HD-MEL (CR) |
| Control-1 | F | 74 | 4.4 |  | |
| Control-2 | F | 76 | 6.3 |  |  |
| Control-3 | F | 63 | 8.2 |  |  |
| Control-4 | F | 37 | 11.0 |  |  |
| Control-5 | M | 78 | 8.6 |  |  |
| Control-6 | M | 44 | 10.5 |  |  |

CKD: Chronic Kidney Disease, CR: Complete Response, F: Female, HD-MEL: High dose Melphalan, M: Male, MM: Multiple Myeloma,

NCC: Nucleated Cell Count, VGPR: Very Good Partial Response, PD: Progressive Disease. *Normal range of NCC in bone marrow is 10-25 x10^4^/μL
